# Purification of crime scene DNA extracts using centrifugal filter devices

**DOI:** 10.1186/2041-2223-4-8

**Published:** 2013-04-24

**Authors:** Lina Norén, Ronny Hedell, Ricky Ansell, Johannes Hedman

**Affiliations:** 1 Swedish National Laboratory of Forensic Science (SKL), Linköping, SE, 581 94, Sweden; 2 Department of Mathematical Sciences, Chalmers University of Technology, Göteborg, SE, 412 96, Sweden; 3 Department of Physics, Chemistry and Biology (IFM), Linköping University, Linköping, SE, 581 83, Sweden; 4 Division of Applied Microbiology, Lund University, Lund, SE, 221 00, Sweden

**Keywords:** Amicon Ultra, DNA purification, DNA recovery, Forensic DNA analysis, Microsep, PCR inhibition, PCR inhibitors

## Abstract

**Background:**

The success of forensic DNA analysis is limited by the size, quality and purity of biological evidence found at crime scenes. Sample impurities can inhibit PCR, resulting in partial or negative DNA profiles. Various DNA purification methods are applied to remove impurities, for example, employing centrifugal filter devices. However, irrespective of method, DNA purification leads to DNA loss. Here we evaluate the filter devices Amicon Ultra 30 K and Microsep 30 K with respect to recovery rate and general performance for various types of PCR-inhibitory crime scene samples.

**Methods:**

Recovery rates for DNA purification using Amicon Ultra 30 K and Microsep 30 K were gathered using quantitative PCR. Mock crime scene DNA extracts were analyzed using quantitative PCR and short tandem repeat (STR) profiling to test the general performance and inhibitor-removal properties of the two filter devices. Additionally, the outcome of long-term routine casework DNA analysis applying each of the devices was evaluated.

**Results:**

Applying Microsep 30 K, 14 to 32% of the input DNA was recovered, whereas Amicon Ultra 30 K retained 62 to 70% of the DNA. The improved purity following filter purification counteracted some of this DNA loss, leading to slightly increased electropherogram peak heights for blood on denim (Amicon Ultra 30 K and Microsep 30 K) and saliva on envelope (Amicon Ultra 30 K). Comparing Amicon Ultra 30 K and Microsep 30 K for purification of DNA extracts from mock crime scene samples, the former generated significantly higher peak heights for rape case samples (*P*-values <0.01) and for hairs (*P*-values <0.036). In long-term routine use of the two filter devices, DNA extracts purified with Amicon Ultra 30 K were considerably less PCR-inhibitory in Quantifiler Human qPCR analysis compared to Microsep 30 K.

**Conclusions:**

Amicon Ultra 30 K performed better than Microsep 30 K due to higher DNA recovery and more efficient removal of PCR-inhibitory substances. The different performances of the filter devices are likely caused by the quality of the filters and plastic wares, for example, their DNA binding properties. DNA purification using centrifugal filter devices can be necessary for successful DNA profiling of impure crime scene samples and for consistency between different PCR-based analysis systems, such as quantification and STR analysis. In order to maximize the possibility to obtain complete STR DNA profiles and to create an efficient workflow, the level of DNA purification applied should be correlated to the inhibitor-tolerance of the STR analysis system used.

## Background

Biological samples from crime scenes are heterogeneous, as any human cell type deposited on any material or surface can be recovered and used as evidence. Forensic DNA analysis is limited by the size, quality and purity of these samples. Efficient sample treatment protocols are needed to release and concentrate the nucleic acids and remove PCR-inhibitory compounds, thus maximizing the analytical success rate [1,2].

Sample treatment generally includes i) eluting cells from evidence item, swab or mini-tape, ii) cell lysis, and iii) DNA purification. In this process, there is generally a trade-off between yield and purity. Physical separation of cells from the background material prior to lysis, for example, by laser microdissection or differential centrifugation methods [3,4], can improve sample purity. However, these methods are timeconsuming, laser microdissection is very costly and differential centrifugation generally gives poor recovery rates (below 50%) [5]. Direct lysis is more straightforward and generates higher yields, and has therefore become the most common approach in forensics [6]. Cell lysis can be chemical (for example, using detergents), enzymatic (for example, proteinase K treatment), physical (for example, heating) or mechanical (for example, bead-beating). Direct lysis involves the obvious risk of co-extracting disturbing substances with physicochemical properties similar to DNA. Extensive DNA purification can therefore be needed to generate PCR-compatible extracts [7-9]. DNA purification, however, inevitably leads to DNA loss [10,11]. The level of loss is dependent on both sample type and purification method. Recovery rates spanning from 10 to 85% have been reported when comparing different methods for a certain sample type [10].

Post-extraction DNA purification of crime scene samples is generally performed using kits based on silica-coated magnetic beads or silica membranes in manual or automated protocols [12,13] or applying centrifugal filter devices [8,14,15]. Centrifugal filter devices, or microdialysis, have been applied in forensics since the early days of PCR-based DNA analysis [14].

Lately, the forensic application of the Amicon Ultra (Millipore, Billerica, MA, USA) filter device has been reported in several studies, for purification as well as for concentration of DNA extracts [16-20]. However, there is a lack of studies investigating the recovery rate and general performance of this and other centrifugal devices for common crime scene sample types. The recent introduction of new short tandem repeat (STR) DNA typing kits with increased PCR inhibitor tolerance [21,22] also make it relevant to update the view on DNA purification. We have evaluated the recovery rate and purification capacity of the centrifugal filter devices Amicon Ultra 30 K and Microsep 30 K (Pall, Port Washington, NY, USA) and compared their respective performance in long-term routine use.

## Methods

Amicon Ultra 30 K and Microsep 30 K were evaluated using dilution series of extracted DNA and mock crime scene DNA extracts from various sample types and extraction procedures. The non-purified DNA extracts were first quantified and in some cases STR-analyzed (see below) and used as references for calculation of recovery rates. Each extract was split between the two devices, Amicon Ultra 30 K and Microsep 30 K, and purified in parallel to achieve the most accurate comparison regarding recovery rate and general performance. Additionally, the performance of the two filter devices was compared in long-term use in routine casework. In total, 7,869 casework DNA extracts were evaluated with respect to level of PCR inhibition and STR results, of which 4,883 were purified using Amicon Ultra 30 K and 2,986 using Microsep 30 K.

### Preparation of DNA for investigation of recovery rates

Pure DNA was prepared for the recovery rate study. DNA extracted from whole blood using BioRobot M48 (Qiagen, Hilden, Germany) was quantified (see below) and diluted to 2.0, 0.5, and 0.2 ng/μL. Five 200 μL replicates per concentration and type of filter device (Amicon Ultra 30 K and Microsep 30 K) were analyzed. One wash cycle was applied in the filter purification (see below). All extracts were quantified before and after centrifugal filter purification.

### Preparation of DNA from mock crime scene stains

DNA extracts were prepared for various mock crime scene sample types. All extractions were performed using Chelex [23], except where other methods are indicated. The samples were quantified and STR-analyzed (see below). For blood on denim, blood (20 μL of diluted blood corresponding to 2 μL of whole blood) was placed on 0.5 × 0.5 cm pieces of denim fabric and left to dry (three samples). For blood on paper, 10 samples of blood on kitchen paper (30 μL of diluted blood corresponding to 3 μL whole blood on 0.5 × 0.5 cm pieces of paper) were left to dry before performing organic (phenol) extraction [24]. For hair, 10 anagen hairs were cut 0.5 cm from the root and each hair was placed in a 1.5 mL microcentrifuge tube with hair buffer for Chelex extraction. For rape case samples, 24 samples with both semen (1 μL of semen in 30 μL water) and saliva (80 μL of mouth rinse, from 5 mL of tap water rinsed for one minute, added to mimic the epithelial fraction of sex-crime samples) were prepared on cotton swabs and left to dry before extraction using Chelex-based differential lysis extraction, generating 24 semen and 24 epithelial cell fractions. For saliva on envelopes, the envelopes were sealed with saliva and left to dry, and 1 × 1.5 cm pieces of the adhesive edges were cut and divided into six equally sized strips prior to extraction (three samples). For touch stains, 10 pieces of mini-tape were used for collection of cells from a pair of tights that had been worn for a couple of days prior to sample collection [25]. For inhibitory samples, DNA (100 μL of 0.2 ng/μL DNA, from the recovery rate study, see above) was mixed with moist snuff extract (100 μL), corresponding to one portion of moist snuff. Three replicates were purified using Amicon Ultra 30 K. For the moist snuff samples, one wash cycle was applied in filter purification.

### Centrifugal filter purification

Centrifugal filter purification was performed using Amicon Ultra 30 K and Microsep 30 K following the manufacturer’s recommendations [26,27]. In short, DNA extract and 1 × TE^-4^-buffer were added to the devices giving a total volume of 2 mL for Amicon Ultra 30 K and 4 mL for Microsep 30 K, followed by centrifugation (4000 × g for 10 minutes for Amicon Ultra 30 K and 4000 × g for 15 minutes for Microsep 30 K). Then, the filter collection tubes were emptied and the devices refilled to 2 or 4 mL 1 × TE^-4^-buffer for a second centrifugation step/wash cycle, unless otherwise noted. DNA was eluted by reverse spinning of the filter (1000 × g for 2 minutes for Amicon Ultra 30 K and 1000 × g for 3 minutes for Microsep 30 K). Following centrifugal dialysis, the purified extracts were diluted to the input extract volume with 1 × TE^-4^-buffer to ensure comparable amplification conditions.

### DNA quantification and estimation of PCR inhibition

DNA quantification was performed using the Quantifiler Human kit and ABI7300 real-time PCR instrument (Life Technologies, Carlsbad, CA, USA) following the manufacturer’s recommendations [28], with the exception that the standard curve was expanded to 0.006 ng/μL DNA. The Quantifiler Human internal PCR control (IPC) was used to estimate PCR inhibition in the casework samples. The normal IPC quantification cycle (Cq) value was defined as the average of IPC Cq:s from amplification of standard DNA 0.006 to 1.85 ng/μL plus three times the standard deviation. Samples with Cq values above this normal IPC Cq were considered inhibitory.

### STR analysis

Multiplex STR amplification and DNA profile generation was conducted using the PowerPlex ESX 16 kit (Promega, Madison, WI, USA), ABI9700 thermal cycler, and ABI Prism 3130xl capillary electrophoresis instrument (3 kV, 5 s injection time) with the software ABI Prism 3130xl Data Collection Software and GeneMapper ID v 3.2.1 (Life Technologies) following the manufacturer’s recommendations [29].

### Data analysis and statistical methods

Recovery rates were calculated from the mean DNA concentrations of five replicates. The systematic difference between DNA purification methods in the recovery study was assessed by the Welch two-sample *t*-test [30,31]. Electropherogram quality is presented as i) detected alleles, that is, true allelic peaks over 50 relative fluorescence units (rfu); ii) total sum of STR peak heights, normalized against the sample volume applied in PCR (intensity); iii) mean local balance, or mean heterozygote balance, that is, the height of the smaller allele of a heterozygote couple divided by the height of the larger allele (intra-locus balance), and iv) normalized Shannon entropy (inter-locus balance) [32,33]. These quantities were computed for each sample, and then summarized for each sample type. For measurement i) the results were summarized as the fraction of detected alleles. For ii) to iv) the average values were computed for each sample type. In addition, as each extract volume was divided equally between the devices when different extraction methods were examined, pairwise comparisons between results could also be performed. This was done for measure ii) to iv) for each sample type, as well for the DNA quantification results, by two types of statistical tests for the systematic difference between the methods. First a binomial test, only considering which method gave the highest result for each pair of samples [34,35]. Secondly, a pairwise *t*-test [31,36] assuming approximately normally distributed differences, either on the linear or logarithmic scale. Differences with *P*-values below 0.05 for both tests were considered significant.

## Results and discussion

### Recovery rate

Following centrifugal filter purification, Microsep 30 K retained 14 to 32% of the input DNA for the different amounts tested, whereas Amicon Ultra 30 K retained 62 to 70% (Table 1). The DNA losses were statistically significant for both filter devices, and Amicon Ultra 30 K retained significantly more DNA compared with Microsep 30 K (*P*-values <0.01) (see Additional file 1: Table S1). In an additional experiment applying Amicon Ultra 30 K, an increase in the number of wash cycles appeared to further reduce the amount of DNA: one extra cycle lowered the DNA amount by another 30%, and two extra cycles by a total of 39%, compared to the standard washing procedure (results not shown).

**Table 1:** DNA recovery rates following centrifugal filter purification using Amicon Ultra 30 K and Microsep 30 K

**Starting DNA concentration (ng/μL)**	**Recovery rate, Amicon Ultra 30 K (n = 5)**	**Recovery rate, Microsep 30 K (n = 5)**
**0.2**	62%	18%
**0.5**	69%	14%
**2.0**	70%	32%

The DNA loss of centrifugal filter purification is likely due to attachment to tube walls or filter parts [1]. The size of the membrane pores should ensure that only smaller molecules pass through, retaining the larger DNA molecules. A pore size of 30 K should retain DNA molecules with nominal molecular weights above 30,000, corresponding to double-stranded DNA of around 50 base pairs. Here, increasing the number of wash cycles lowered the DNA recovery, suggesting that more thorough washing/centrifugation makes the DNA bind tighter to and/or get trapped within the filter. Therefore, the trade-off between purity and recovery should be taken into consideration when deciding on the number of applied wash cycles.

### Purification of mock crime scene DNA extracts

Comparing Amicon Ultra 30 K and Microsep 30 K for purification of DNA extracts from the various mock crime scene sample types, the former generated electropherograms with significantly higher STR total peak heights for the mock rape case samples and hairs (*P*-value <0.01 and <0.036 respectively) (Table 2 and Additional file 1: Table S2). For touch stains on mini-tapes and blood on kitchen paper the filter devices generated non-significant peak height differences. The differences in number of detected alleles, as well as intra- and inter-loci balance of electropherograms, were minor for all sample types (Table 2 and Additional file 1: Table S2).

**Table 2:** Centrifugal filter purification of mock crime scene DNA extracts using Amicon Ultra 30 K and Microsep 30 K

**Sample type**	**DNA purification**	**Average DNA concentration (ng/μL) with CV (%)**	**Detected STR alleles (>50 rfu)**	**Average of total sum of STR peak heights (rfu)**^**a **^**with CV (%)**	**Average of intra-locus balance (0 to 1)**^**b**^	**Average of inter-loci balance (0 to 1)**^**c**^
**Blood on denim (n = 3)**	None	0.46 (27%)	96%	30,776 (20%)	0.83	0.90
	Microsep 30 K	0.22(12%)	100%	42,113 (10%)	0.87	0.96
	Amicon Ultra 30 K	0.27(15%)	96%	51,596 (38%)	0.84	0.95
**Blood on kitchen paper (n = 10)**	None	0.32 (79%)	N/A	N/A	N/A	N/A
	Microsep 30 K	0.23 (83%)	100%	20,230 (78%)	0.89	0.99
	Amicon Ultra 30 K	0.23 (85%)	100%	21,115 (96%)	0.90	0.99
**Hair (n = 10)**	None	0.66 (77%)	N/A	N/A	N/A	N/A
	Microsep 30 K	0.39 (77%)	98%	32,135 (77%)	0.88	0.98
	Amicon Ultra 30 K	0.72 (55%)	100%	62,670 (56%)	0.93	0.99
**Rape case samples, semen fraction (n = 24)**	None	1.10 (34%)	N/A	N/A	N/A	N/A
	Microsep 30 K	0.56 (43%)	100%	57,088 (37%)	0.89	0.98
	Amicon Ultra 30 K	0.86 (25%)	100%	85,441 (23%)	0.91	0.99
**Rape case samples, epithelial fraction (n = 24)**	None	1.09 (51%)	N/A	N/A	N/A	N/A
	Microsep 30 K	0.68 (39%)	100%	33,498 (47%)	0.87	0.97
	Amicon Ultra 30 K	0.89 (30%)	100%	42,508 (30%)	0.88	0.97
**Saliva on envelope (n = 3)**	None	0.083 (22%)	100%	53,754 (14%)	0.90	0.98
	Microsep 30 K	0.050 (8%)	100%	43,682 (10%)	0.91	0.99
	Amicon Ultra 30 K	0.054 (17%)	100%	63,168 (6%)	0.88	0.99
**Touch stains (mini-tape) (n = 10)**	None	0.023 (71%)	N/A	N/A	N/A	N/A
	Microsep 30 K	0.027 (55%)	95%	11,281 (73%)	0.74	0.96
	Amicon Ultra 30 K	0.024 (59%)	97%	12,207 (77%)	0.76	0.96

^a^Total sum of peak heights have been normalized against the sample volume in PCR. ^b^Mean local balance, that is, the mean of the heterozygote balances in all markers of the electropherograms, was used for calculation of intra-locus balance. ^c^Normalized Shannon entropy [32,33] was used to calculate inter-locus balance. *CV*, Coefficient of variation; *rfu*, Relative fluorescence units.

The average DNA concentrations were slightly lowered by filter purification compared with the non-purified extracts, except for touch stains and hair (Table 2). However, for both Amicon Ultra 30 K and Microsep 30 K the calculated DNA loss was only significant for the rape case samples (*P*-values <0.05) (see Additional file 1: Table S2). From the recovery rate study it is obvious that filter purification leads to substantial DNA loss. There, the recovery rates were determined applying highly purified DNA. When analyzing impure mock crime scene samples, the removal of PCR-inhibitory compounds counteracts some of the DNA loss through improved amplifiability, leading to smaller differences in the measured DNA concentrations. For ten of the rape case samples and two samples of blood on kitchen paper, Quantifiler Human qPCR generated negative results with the crude extracts due to strong PCR inhibition (IPC not detected, results not shown). All of these samples generated usable DNA concentrations following purification using both Amicon Ultra 30 K and Microsep 30 K (rape case samples: 0.69 to 1.54 ng/μL for Amicon Ultra 30 K, 0.46 to 1.19 ng/μL for Microsep 30 K; blood on kitchen paper: 0.04 and 0.09 for Amicon Ultra 30 K, 0.09 to 0.10 ng/μL for Microsep 30 K).

Blood on denim and saliva on envelopes are particularly difficult sample types, containing several known PCR inhibitors such as lactoferrin and hematin in blood [37], indigo dye in denim [9] and cellulose in paper [38]. Here, both Amicon Ultra 30 K and Microsep 30 K produced complete or almost complete DNA profiles for theses sample types (Table 2). Despite the DNA loss from filter purification, Amicon Ultra 30 K generated increased peak heights, although non-significant compared to the non-purified extracts (Table 2 and Additional file 1: Table S2). Microsep 30 K provided increased peak heights for blood on denim but slightly lowered peak heights for saliva on envelopes. Neither of these differences were statistically significant. However, it is clear that the removal of PCR-inhibitory substances through filtration improves the amplifiability. Had this not been the case, filter purification would have led to decreased allelic peak heights.

**Figure 1:**
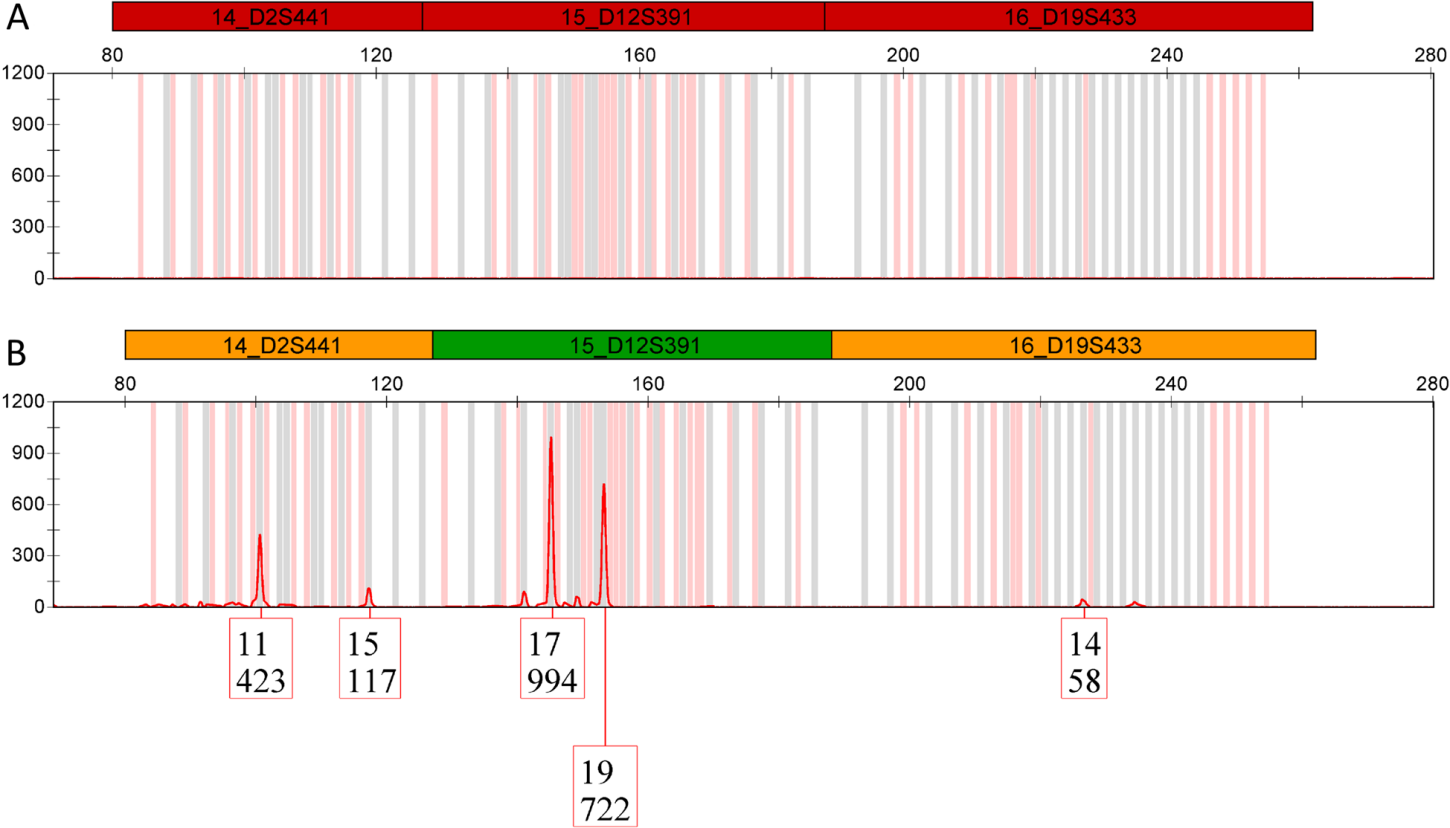
Electropherograms showing PowerPlex ESX 16 amplification (red panel) of moist snuff extract A) before and B) after Amicon Ultra 30 K centrifugal filter purification.

Amicon Ultra 30 K was also applied for purification of moist snuff extracts. The device enabled detection of 52% of the STR alleles (47 of 90, three replicates), whereas the non-purified extract failed to generate a single allele (Figure 1). However, the poor intra- and inter-loci balances show that even after purification the extracts are inhibitory.

### Comparison of Amicon Ultra 30 K and Microsep 30 K in long-term routine use

Centrifugal filter purification using Amicon Ultra 30 K was introduced in routine casework at the laboratory in 2011, replacing Microsep 30 K for purification of impure extracts and for concentrating extracts. When studying the outcome of long-term use of the two filter devices, it was found that extracts purified with Amicon Ultra 30 K were considerably less PCR-inhibitory in Quantifiler Human analysis compared to Microsep 30 K (Table 3). The differences were greatest for organically extracted samples such as bloodstains (11% of samples purified with Amicon Ultra 30 K caused elevated IPC Cq values, compared with 52% for Microsep 30 K), and rape case samples (16% Amicon Ultra 30 K and 41% for Microsep 30 K). For touch stains on mini-tapes and hairs, the two methods generated extracts of more similar purity with a slight advantage for Amicon Ultra 30 K. For STR typing with PowerPlex ESX16 the two filter purification methods produced electropherograms of comparable quality concerning the fraction of negative DNA profiles and number of usable single-donor profiles (results not shown). This implies that Quantifiler Human and PowerPlex ESX 16 are not correlated with respect to inhibitor-tolerance, and that the latter system is more resistant to some of the extraneous substances present in common crime scene DNA extracts.

**Table 3:** Purity of extracts in routine casework using Microsep 30 K and Amicon Ultra 30 K

**Sample type**	**Inhibition following Microsep 30 K purification (1 May 2011 to 31 August 2011)**	**Inhibition following Amicon Ultra 30 K purification (1 October 2011 to 29 February 2012)**
**Rape case samples**	41% (n = 1,432)	16% (n = 2,094)
**Hairs**	31% (n = 94)	26% (n = 130)
**Touch stains on mini-tape**	43% (n = 1,439)	26% (n = 2,641)
**Organically extracted samples**	52% (n = 21)	11% (n = 18)

Crime scene DNA extracts are defined as PCR-inhibitory if they generate a shift of the Quantifiler Human IPC Cq compared to the normal value for pure reactions.

PCR-inhibitory effects from various compounds can differ between different analysis systems due to differences in sample-to-reaction-volume ratio and the type of DNA polymerase used [32,39]. Thus, a specific substance can inhibit Quantifiler Human but not PowerPlex ESX 16, or vice versa. Pure extracts will improve the consistency between the qPCR-based quantification system and the STR analysis system, or between different STR/Y-STR analysis systems, thereby streamlining analysis by reducing the risk of over- or underestimating the amount of DNA available for profiling.

## Conclusions

Extensive DNA purification can be necessary for successful DNA profiling and consistency between different PCR-based analysis systems. However, purification also leads to DNA loss. The recovery depends on the contents of the specific sample and the employed purification procedure [10]. We found that the two commonly used filter purification devices Amicon Ultra 30 K and Microsep 30 K give pronounced recovery rate differences (62% to 70%, and 14% to 32%, respectively). However, in crime scene DNA sample analysis this DNA loss can be counteracted by the improved amplifiability for the purified extract.

The use of STR analysis kits and DNA polymerase-buffer systems with improved PCR inhibitor tolerance has pushed the limit for successful forensic DNA analysis and reduced the need for highly purified DNA [11,21,22,40]. To reduce DNA loss and workload, we recommend laboratories to carefully review and possibly update their DNA purification methods when implementing new, inhibitor-tolerant analysis kits. However, there will always be cases where extensive DNA purification is necessary to enable amplification, such as seen here for the moist snuff samples. Additionally, pure DNA extracts are more stable in long-term storage. High levels of proteins such as nucleases and ions catalyzing these nucleases inevitably lead to degradation of DNA even at sub-zero temperatures.

In total, Amicon Ultra 30 K performed better than Microsep 30 K with higher DNA recovery and more efficient removal of inhibitory substances. The different performances of the filter devices are likely caused by the quality of the filters and plastic wares, for example, their DNA binding properties.

We conclude that centrifugal filter purification of crime scene DNA extracts leads to DNA loss, but the elevated purity can counteract this loss through improved amplifiability. Higher purity also provides better consistency between different PCR-based analysis systems, such as qPCR-based quantification and STR analysis. Amicon Ultra 30 K is suitable for purification of crime scene DNA extracts from various origins. In order to maximize the possibility to obtain complete STR DNA profiles and create an efficient workflow, the level of DNA purification applied should be correlated to the inhibitor-tolerance of the STR analysis system used.

## Abbreviations

Cq: Quantification cycle; IPC: Internal PCR control; PCR: Polymerase chain reaction; rfu: Relative fluorescence units; STR: Short tandem repeat.

## Competing interests

The authors declare that they have no competing interests.

## Authors’ contributions

JH outlined the study with support from RA. JH and LN designed the experiments with support from RH. LN performed the experiments. RH performed statistical calculations. JH and RA wrote the manuscript, with help from LN and RH. All authors read and approved the final version of the manuscript.

## Supplementary Material

Additional file 1 **Table S1.** Statistical tests for the systematic difference in DNA concentration (ng/μL) between DNA purification methods in the recovery study. **Table S2**. Statistical tests for the systematic difference between DNA purification methods for the various mock crime scene DNA extracts, based on pairwise comparison of results. Click here for file
