# Supplementary material for: Purification of crime scene DNA extracts using centrifugal filter devices
Source: Investig Genet. 2013 Apr 24;4:8. doi: 10.1186/2041-2223-4-8 (PMC3640930; doi:10.1186/2041-2223-4-8)
Supplement: Additional file 1 — Table S1. Statistical tests for the systematic difference in DNA concentration (ng/μL) between DNA purification methods in the recovery study. Table S2. Statistical tests for the systematic difference between DNA purification methods for the various mock crime scene DNA extracts, based on pairwise comparison of results. [file 2041-2223-4-8-S1.docx]

**ADDITIONAL FILE 1**

**Purification of crime scene DNA extracts using centrifugal filter devices**

L. Norén, R. Hedell, R. Ansell, J. Hedman

Contents: Two tables containing statistical test results for the evaluation of the centrifugal filter devices Amicon Ultra 30K and Microsep 30K

Additional file 1: Table S1. Statistical tests for the systematic difference in DNA concentration (ng/µL) between DNA purification methods in the recovery experiment.

| **Starting DNA concentration (ng/µL)** | **DNA purification methods in comparison** | **Difference in mean DNA concentration (ng/µL) (N=5)** | **p-value^a^** |
| --- | --- | --- | --- |
| 0.2 | None vs. Amicon Ultra 30K | 0.05 | 0.0098 |
|  | None vs. Microsep 30K | 0.12 | 0.00059 |
|  | Amicon Ultra 30K vs. Microsep 30K | 0.06 | 0.00046 |
| 0.5 | None vs. Amicon Ultra 30K | 0.15 | 7.8e-05 |
|  | None vs. Microsep 30K | 0.43 | 0.00082 |
|  | Amicon Ultra 30K vs. Microsep 30K | 0.27 | 0.0018 |
| 2 | None vs. Amicon Ultra 30K | 0.65 | 0.0038 |
|  | None vs. Microsep 30K | 1.48 | 1.8e-07 |
|  | Amicon Ultra 30K vs. Microsep 30K | 0.83 | 0.0011 |

a) P-value calculated using Welch’s two sample t-test [[30](#_ENREF_30), [31](#_ENREF_31)].

Additional file 1: Table S2. Statistical tests for the systematic difference between DNA purification methods, based on pairwise comparison of results. For each comparison there are results from two tests. Bold numbers indicate significant differences (p-values below 0.05).

| **Sample type** | **DNA purification methods in comparison** | **DNA concentration, test values and p-values^a,b^** | **Total STR peak height, test values and p-values ^a,c^** | **Intra-locus balance, test values and p-values ^a,d^** | **Inter-loci balance, test values and p-values ^a,e^** |
| --- | --- | --- | --- | --- | --- |
| **Blood on denim**  **(N=3)** | None vs. Amicon Ultra 30K | 1, p: 0.25;  0.5, p: 0.14 | 0, p: 0.25;  -0.47, p: 0.11 | 0.33, p: 1;  -0.012, p: 0.84 | 0.33, p: 1;  -0.046, p: 0.2 |
|  | None vs. Microsep 30K | 1, p: 0.25;  0.7, p: **0.024** | 0, p: 0.25;  -0.32, p: **0.033** | 0, p: 0.25;  -0.044, p: **0.026** | 0, p: 0.25;  -0.056, p: **0.008** |
|  | Amicon Ultra 30K vs. Microsep 30K | 0.67, p: 1;  0.2, p: 0.18 | 0.67, p: 1;  0.15, p: 0.56 | 0.67, p: 1;  -0.032, p: 0.58 | 0.67, p: 1;  -0.01, p: 0.76 |
| **Blood on kitchen paper (N=10)** | None vs. Amicon Ultra 30K | 0.62, p: 0.73;  0.034, p: 0.78 | N/A | N/A | N/A |
|  | None vs. Microsep 30K | 0.75, p: 0.29;  0.077, p: 0.72 | N/A | N/A | N/A |
|  | Amicon Ultra 30K vs. Microsep 30K | 0.4, p: 0.75;  -0.053, p: 0.66 | 0.4, p: 0.75;  -0.024, p: 0.81 | 0.7, p: 0.34;  0.0037, p: 0.63 | 0.5, p: 1;  -0.00069, p: 0.74 |
| **Hair (N=10)** | None vs. Amicon Ultra 30K | 0.5, p: 1;  -0.28, p: 0.27 | N/A | N/A | N/A |
|  | None vs. Microsep 30K | 0.6, p: 0.75;  0.49, p: 0.22 | N/A | N/A | N/A |
|  | Amicon Ultra 30K vs. Microsep 30K | 0.9, p: **0.021**;  0.77, p: **0.012** | 1, p: **0.002**;  0.81, p: **0.035** | 1, p: **0.002**;  0.052, p: 0.057 | 0.9, p: **0.021**;  0.014, p: **0.028** |
| **Rape case samples, semen fraction (N=24)** | None vs. Amicon Ultra 30K | 0.77, p: **0.017**;  0.19, p: **0.046** | N/A | N/A | N/A |
|  | None vs. Microsep 30K | 0.86, p: **0.00086**;  0.67, p: **1.1e-05** | N/A | N/A | N/A |
|  | Amicon Ultra 30K vs. Microsep 30K | 0.79, p: **0.0066**  0.49, p: **7.1e-05** | 1, p: **1.2e-07**;  0.44, p: **1.2e-08** | 0.67, p: 0.15;  0.015, p: **0.038** | 0.58, p: 0.54;  0.0033, p: **0.034** |
| **Rape case samples, epithelial fraction (N=24)** | None vs. Amicon Ultra 30K | 0.79, p: 0.057;  -0.044, p: 0.89 | N/A | N/A | N/A |
|  | None vs. Microsep 30K | 0.86, p: **0.013**;  0.33, p: 0.16 | N/A | N/A | N/A |
|  | Amicon Ultra 30K vs. Microsep 30K | 0.96, p: **3e-06**;  0.31, p: **3.8e-05** | 0.83, p: **0.0015**;  0.3, p: **0.00015** | 0.67, p: 0.15;  0.012, p: 0.11 | 0.38, p: 0.31;  0.00019, p: 0.85 |
| **Saliva on envelope (N=3)** | None vs. Amicon Ultra 30K | 1, p: 0.25;  0.43, p: 0.093 | 0.33, p: 1;  -0.17, p: 0.27 | 1, p: 0.25;  0.015, p: 0.15 | 0, p: 0.25;  -0.01, p: **0.014** |
|  | None vs. Microsep 30K | 1, p: 0.25;  0.48, p: **0.048** | 0.67, p: 1;  0.2, p: 0.25 | 0.33, p: 1;  -0.0072, p: 0.63 | 0.33, p: 1;  -0.0097, p: 0.2 |
|  | Amicon Ultra 30K vs. Microsep 30K | 0.67, p: 1;  0.057, p: 0.41 | 1, p: 0.25;  0.37, p: **0.005** | 0.33, p: 1;  -0.022, p: 0.37 | 0.33, p: 1;  0.00053, p: 0.93 |
| **Touch stains (mini-tape) (N=10)** | None vs. Amicon Ultra 30K | 0.4, p: 0.75;  -0.098, p: 0.23 | N/A | N/A | N/A |
|  | None vs. Microsep 30K | 0.33, p: 0.51;  -0.17, p: 0.28 | N/A | N/A | N/A |
|  | Amicon Ultra 30K vs. Microsep 30K | 0.44, p: 1;  -0.095, p: 0.36 | 0.7, p: 0.34;  0.051, p: 0.48 | 0.7, p: 0.34;  0.019, p: 0.32 | 0.7, p: 0.34;  0.0052, p: 0.33 |

a) Test 1: Test value shows the fraction of samples with a higher result for the first method than for the second method in comparison. A test value above 0.5 indicate a potential support for the first method in comparison while a test value below 0.5 indicate a potential support for the second method in comparison. P-value is from the binomial test [[34](#_ENREF_34), [35](#_ENREF_35)]. Test 2: Test value shows the average of differences, either on the linear (additive difference) or logarithmic (multiplicative difference) scale. A positive number indicate a potential support for the first method in comparison while a negative number indicate a potential support for the second method in comparison. P-value is from the pairwise t-test [[31](#_ENREF_31), [36](#_ENREF_36)].

b) Multiplicative difference assumed.

c) Multiplicative difference assumed.

d) Additive difference assumed. The mean of the heterozygote balances in all markers of the EPGs, was used for calculation of intra-locus balance.

e) Additive difference assumed. Normalized Shannon entropy [[32](#_ENREF_32), [33](#_ENREF_33)] was used to calculate inter-locus balance.
